# Torque Ripple Suppression of Brushless DC Motor Drive System Based on Improved Harmonic Injection Active Disturbance Rejection Control

**DOI:** 10.3390/s22031069

**Published:** 2022-01-29

**Authors:** Jinglun He, Changxiang Yan, Xiaodong Wang

**Affiliations:** 1 Changchun Institute of Optics, Fine Mechanics and Physics, Chinese Academy of Sciences, Changchun 130033, China; hejinglun666@163.com (J.H.); wangxd@ciomp.ac.cn (X.W.); 2 University of Chinese Academy of Sciences, Beijing 100049, China; 3 Center of Materials Science and Optoelectrics Engineering, University of Chinese Academy of Sciences, Beijing 100049, China

**Keywords:** active disturbance rejection control (ADRC), brushless DC motor, harmonic injection, torque ripple suppression

## Abstract

The positioning accuracy and speed stability of the brushless DC motor (BLDC motor), as the drive element of the optomechanically scanned system (OMSS), are closely interrelated to the final imaging quality of the system. Active disturbance rejection control (ADRC) with strong anti-interference ability, fast response and good robustness is one of the extensively used control strategies. However, the performance of ADRC working in a complicated environment will be limited due to the controller structure, parameter tuning and the influence of multi-source nonlinear disturbance. Therefore, an improved ADRC method is proposed, which can switch between ‘point-to-point control mode’ and ‘stable speed control mode’ according to the system requirements. To further suppress the torque ripple and improve the control performance of the system, an improved harmonic injection scheme is added, and the parameters of improved ADRC are tuned by a slime mould algorithm based on a Levy flight operator (LF-SMA). The stability of the proposed ADRC is proved by Lyapunov stability theory. The experimental results show that the proposed control scheme could be available to reduce the torque ripple of the system.

## 1. Introduction

In the past few years, brushless DC (BLDC) motors are widely used to drive the optical mirror rotation in the optomechanically scanned system (OMSS) of the airborne scanning hyperspectral imaging spectrometer (ASHIS) [1,2,3]. Owing to the advantages of simple structure, good performance of torque-speed characteristic, long life time, high efficiency and low cost, etc, the OMSS driven by a BLDC motor has great convenience for structure and optical design. Nevertheless, affected by the defects of the BLDC motor (e.g., torque ripple) and the poor working environment of the ASHIS, it is arduous for the control schemes (e.g., proportional integral derivative (PID) control) to achieve desirable control performance which rely on reasonably precise model. Especially for the OMSS, the control performance directly affects the final imaging quality.

To improve the positioning accuracy and speed stability of the BLDC motor servo system, the advanced PID controller is still widely used because of its convenience and effectiveness, such as cascade control [4], feedforward control [5], fuzzy control [6,7]. However, due to the indeterminate complex structure and the nonlinear coupling of the system, the performance of the PID controller is still limited. The cascade control can improve the control performance to a certain extent, but the system needs more sensors at the same time, which increases the cost of the system and the difficulty of tuning parameters accordingly. A feed-forward controller often requires the accuracy of the system model, which is a great challenge for complex systems. For the fuzzy PID controller, the appropriate fuzzy rule table can improve the control performance of a certain aspect. However, for relatively complex systems, the dynamic control performance will be reduced to some extent. At the same time, in order to achieve high-precision control performance, it is necessary to construct complex fuzzy rules, which will lead to the expansion of the search scope and the decision-making period. Sometimes, it may even get out of control.

Compared with the PID controller, active disturbance rejection control (ADRC) proposed by Han, which is characterized by strong robustness and anti-interference ability [8], has been extensively applied in many fields. In different application scenarios, some improved ADRC strategies show excellent performance in positioning or speed stability [9,10,11,12]. The ADRC-based control schemes effectively improve system performance and anti-interference ability, but they only focus on position or speed performance, while OMSS needs both. Therefore, the structure of ADRC needs to be improved for the requirements of OMSS. Simultaneously, using a high efficiency parameter tuning algorithm could effectively enhance the control performance [13,14,15].

The ESO of ADRC extended an extra state to observe the total disturbance for disturbance reduction. Therefore, in the complex working environment, due to the coupling of multi-source nonlinear disturbance, the suppression effect of ADRC on the internal disturbance of the BLDC motor ( such as torque ripple ) will be greatly reduced. Furthermore, lowering the value of the electromagnetic torque ripple decreases the levels of vibration and noise, as well as extending the life expectancy of the drive system [16].

In order to achieve better anti-disturbance performance of ADRC, the control scheme in reference [17] makes use of a special state transformation and a dedicated observer capable of reconstructing various types of disturbances, including complex harmonic signals. In reference [18,19], a novel adaptive resonant extended state observer (RESO) is designed to obtain the frequency of the periodic disturbance in tracking signal online. A continuously updated two-tier control action is applied to compensate for the effect of the total disturbance on the output.

Repetitive control (RC) [20,21,22,23,24] and iterative learning control (ILC) [25,26,27,28] are also widely used to suppress the periodic disturbance of the BLDC motor drive system, which often perform well in repetitive tasks. However, for applications that do not strictly meet the repeated execution of the same task under the same conditions, or when there are non-repetitive, random noise or complex nonlinear systems, it often needs to be combined with other control strategies to achieve good performance.

Field oriented control (FOC) is applied in BLDC motor dirve system because of its exllent performance of positioning and torque ripple suppression [29]. However, good performance requires accurate state observation of the control system, which is often attributed to excellent sensors. Adding a torque ripple suppression module can also effectively improve the performance of OMSS. By comparison, one of the most effective approaches is generalized harmonic injection method [30,31,32,33,34,35]. However, most harmonic injection schemes are implemented in rotating reference frame, and the calculation processes are often complicated. At the same time, owing to the limitation of the model, it is difficult to consider the influence of high order components in stator harmonic currents.

Based on the above research status, in order to improve the positioning accuracy and speed stability of the BLDC motor servo system under various uncertainties, and to suppress the torque ripple in BLDC motor, a control scheme based on improved ADRC and harmonic injection method is proposed in this paper.

The main structure of this paper is as follows. Section 1 is the introduction. In Section 2, a detailed mathematical model of the BLDC motor is given, in which the influence of back EMF harmonics is considered. Section 3 introduces the design and parameter tuning method of improved ADRC. Section 4 introduces a torque ripple suppression method based on harmonic injection method under the stationary reference frame. Section 5 gives the experimental results and proves the effectiveness of the control scheme. Finally, some conclusions are drawn in Section 6.

## 2. Mathematical Model of the Brushless DC Motor

For a BLDC motor, the electrical dynamics is described by the following voltage equations.

(1) $$V_{abcs}=L\frac{di_{abcs}}{dt}+R_{s}i_{abcs}+e_{abcs}$$

The mechanical dynamic equation of motor can be expressed as [36]:

(2) $$J\ddot{\theta }+B\dot{\theta }+T_{L}=T_{m}=K_{t}i$$

where *J* is the combined moment of inertia of the system, *B* denotes the damping ratio of the mechanical system (Nms), $T_{L}$ denotes the load torque, $T_{m}$ denotes the combined mechanical torque, $K_{t}$ is the torque constant (Nm/A) and *i* denotes the armature current.

Since $E=K_{e}\dot{\theta }$, and the back EMF constant $K_{e}$ (Vs/rad) equals $K_{t}$ in the SI unit system, the following equation can be obtained by combining Equations (1) and (2).

(3) $$LJ\theta^{\left(3\right)}+\left(LB+RJ\right)\ddot{\theta }+\left(RB+K^{2}\right)\dot{\theta }+\mu =KV$$

After simplification, a single input and single output system can be derived as follows:

(4) $$\theta^{\left(3\right)}=f\left(\dot{\theta },\ddot{\theta },\mu ,t\right)+b_{0}u$$

where *u* and θ are the control signal and the output of the system, respectively, $f\left(\dot{\theta },\ddot{\theta },\mu ,t\right)$ denotes the total disturbance, μ denotes the external disturbance, and $b_{0}$ denotes the control signal coefficient.

## 3. Design and Tuning of Improved Active Disturbance Rejection Control

### 3.1. Control Plant

According to Equation (4), the state equation of the BLDC motor servo system can be expressed as follows:

(5) $$\left\{\begin{array}{c} {\dot{x}}_{1}=x_{2}\\ {\dot{x}}_{2}=x_{3}\\ {\dot{x}}_{3}=x_{4}+b_{0}u \end{array}\right.$$

where $x_{1}$, $x_{2}$ and $x_{3}$ are the system state variable θ, $\dot{\theta }$ and $\ddot{\theta }$, respectively, the state variable $x_{4}$ is extended by the total disturbance of the system.

### 3.2. Design of Improved ADRC

As the driving system of OMSS, BLDC motor servo system has high requirements for stability and accuracy. In order to satisfy the imaging requirements, the system is not only required to achieve ’point-to-point’ position control, but also able to track the given speed stably.

**Figure 1:**
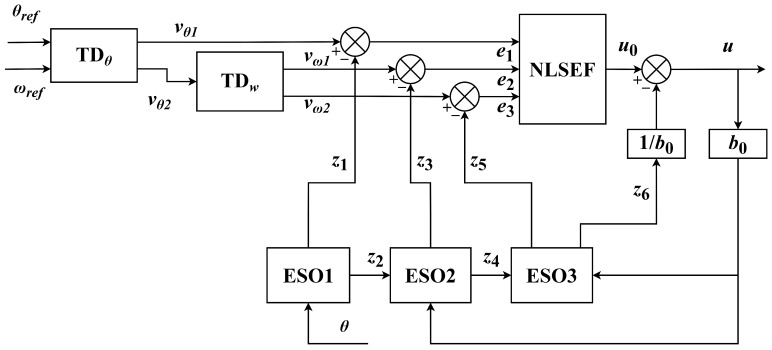
The principle block diagram of the improved ADRC.

According to the requirements and the state Equation (5), a mode switchable ADRC scheme is proposed. One of the advantages of the controller is that it can convert the control mode between ’point-to-point control mode’ and ’stable speed control mode’ according to the control needs without changing the controller parameters. The principle block diagram of the improved ADRC is shown in Figure 1. The input signals can be changed according to the requirements of the system. There are three specific situations: (1) $\theta_{ref}\neq 0,\omega_{ref}=0$; (2) $\theta_{ref}=0,\omega_{ref}\neq 0$; (3) $\theta_{ref}\neq 0,\omega_{ref}\neq 0$. By default, the input signals are positive.

#### 3.2.1. Design of Improved Tracking Differentiator

The main function of the TD is to realize shorter adjustment time, smaller overshoot, stronger stability and better robustness by properly processing the input signals. In our scheme, two first-order TDs are connected in series to process the input signals, which can be equivalent to a second-order TD. The sturcture of the TD can be expressed as follows:

(6) $$TD_{\theta }:\left\{\begin{array}{c} \left(1\right)\theta_{ref}\neq 0,\omega_{ref}=0\left\{\begin{array}{c} e_{\theta }=\nu_{\theta 1}-\theta_{ref}\\ \nu_{\theta 1}=\nu_{\theta 1}+h\nu_{\theta 2}\\ \nu_{\theta 2}=\nu_{\theta 2}+hfosc\left(e_{\theta },\nu_{\theta 2},\sigma_{1},h\right) \end{array}\right.\\ \left(2\right)\theta_{ref}=0,\omega_{ref}\neq 0\left\{\begin{array}{c} e_{\theta }=\left\{\begin{array}{cc} -\varsigma & ,\omega_{ref}>\nu_{\theta 2}\\ \varsigma & ,\nu_{\theta 2}\geq \omega_{ref} \end{array}\right.\\ \nu_{\theta 1}=\nu_{\theta 1}+h\nu_{\theta 2}\\ \nu_{\theta 2}=\left\{\begin{array}{cc} \nu_{\theta 2}+hfosc\left(e_{\theta },\nu_{\theta 2},\sigma_{1},h\right) & ,\omega_{ref}\neq \nu_{\theta 2}\\ \nu_{\theta 2} & ,\omega_{ref}=\nu_{\theta 2} \end{array}\right. \end{array}\right.\\ \left(3\right)\theta_{ref}\neq 0,\omega_{ref}\neq 0\left\{\begin{array}{c} e_{\theta }=\nu_{\theta 1}-\theta_{ref}\\ \nu_{\theta 1}=\nu_{\theta 1}+h\nu_{\theta 2}\\ \nu_{\theta 2}=\left\{\begin{array}{cc} \nu_{\theta 2}+hfosc\left(e_{\theta },\nu_{\theta 2},\sigma_{1},h\right) & ,\omega_{ref}>\nu_{\theta 2}\\ \nu_{\theta 2} & ,\nu_{\theta 2}\geq \omega_{ref}>0 \end{array}\right. \end{array}\right. \end{array}\right.$$

(7) $$TD_{\omega }:\left\{\begin{array}{c} e_{\omega }=\nu_{\omega 1}-\nu_{\theta 2}\\ \nu_{\omega 1}=\nu_{\omega 1}+h\nu_{\omega 2}\\ \nu_{\omega 2}=\nu_{\omega 2}+hfosc\left(e_{\omega },\nu_{\omega 2},\sigma_{2},h\right) \end{array}\right.$$

where $e_{\theta }$ and $e_{\omega }$ are the observer errors, the coefficient ς>0 determines the process of speed adjustment in working mode 2, and *fosc* is the optimal synthesis control function derived from the discrete optimization theory. The expression of *fosc* is as follows:

(8) $$\begin{array}{cc} fosc & =-\left\{\begin{array}{cc} \sigma sign(a) & ,|a|>d\\ \sigma \frac{a}{d} & ,|a|\leq d \end{array}\right.\\ a & =\left\{\begin{array}{cc} \nu_{2}+\frac{a_{0}-d}{2}sign\left(y\right) & ,|y|>d_{0}\\ \nu_{2}+\frac{y}{h} & ,|y|\leq d_{0} \end{array}\right. \end{array}$$

in which d=σh , $d_{0}=\sigma h^{2}$ , $y=e+h\nu_{2}$ , $a_{0}=\sqrt{d^{2}+8\sigma \left|y\right|}$, σ is the coefficient of rate, *h* is the step length, and sign represents the sign function.

#### 3.2.2. Design of Improved Extended State Observer

The main function of the ESO is to observe the total disturbance, which produced by the external unknown part on the control object and the unknown part of the model.

In our scheme, three second-order state observers are used in series to estimate the system state variables accurately. The calculation process of the improved ESO is as follows:

(9) $$\begin{array}{cc} \; & \left\{\begin{array}{c} e_{z1}=z_{1}-\theta \\ z_{1}=z_{1}+h\left(z_{2}-\beta_{1}e_{z1}\right)\\ z_{2}=z_{2}+h\left(-\beta_{2}fal\left(e_{z1},\alpha ,\delta \right)\right) \end{array}\right.\\ \; & \left\{\begin{array}{c} e_{z2}=z_{3}-z_{2}\\ z_{3}=z_{3}+h\left(z_{4}-\beta_{3}e_{z2}\right)\\ z_{4}=z_{4}+h\left(-\beta_{4}fal\left(e_{z2},\alpha ,\delta \right)+b_{0}u\right) \end{array}\right.\\ \; & \left\{\begin{array}{c} e_{z3}=z_{5}-z_{4}\\ z_{5}=z_{5}+h\left(z_{6}-\beta_{5}e_{z3}+b_{0}u\right)\\ z_{6}=z_{6}+h\left(-\beta_{6}fal\left(e_{z3},\alpha ,\delta \right)\right) \end{array}\right. \end{array}$$

(10) $$\begin{array}{c} fal\left(e,\alpha ,\delta \right)=\left\{\begin{array}{cc} \frac{e}{\delta^{\alpha -1}} & ,\left|e\right|\leq \delta \\ {\left|e\right|}^{\alpha }sign\left(e\right) & ,\left|e\right|>\delta \end{array}\right. \end{array}$$

where $fal\left(e,\alpha ,\delta \right)$ is the nonlinear function, $z_{1}$, $z_{3}$ and $z_{5}$ are the observation of the state variable $x_{1}$, $x_{3}$ and $x_{5}$, respectively. The value of α influences the uncertainty of modeling and the adaptability of the disturbance, δ is the linear width of the nonlinear function and $b_{0}$ affects the compensation value.

#### 3.2.3. Nonlinear State Error Feedback Control Law

In NLSEF, the extracted error signals and its tracking signals are combined nonlinearly. The nonlinear function is used to calculate the optimal system input, which enhances the ability and efficiency of the system to eliminate errors. The mathematical expression is as follows:

(11) $$\left\{\begin{array}{c} e_{c1}=\nu_{\theta 1}-z_{1}\\ e_{c2}=\nu_{\omega 1}-z_{3}\\ e_{c3}=\nu_{\omega 2}-z_{5}\\ u_{0}=\zeta_{1}fal\left(e_{c1},\alpha_{1},\delta \right)+\zeta_{2}fal\left(e_{c2},\alpha_{2},\delta \right)+\zeta_{3}fal\left(e_{c3},\alpha_{3},\delta \right)\\ u=u_{0}-\frac{z_{6}}{b_{0}} \end{array}\right.$$

where $e_{1}$, $e_{2}$ and $e_{3}$ are the state error of the system, $u_{0}$ is the error feedback control amount, $\zeta_{1}$ , $\zeta_{2}$ and $\zeta_{3}$ are the gain coefficient. Finally, the estimated value of the total interference needs to be compensated in the generated control signal *u*, and $\frac{z_{6}}{b_{0}}$ represents the compensation value of the total interference.

### 3.3. Stability Analysis

#### 3.3.1. Stability Analysis of Improved TD

The Lyapunov functions of the TD is selected as:

(12) $$V_{1}=\frac{1}{2}e_{\theta }^{2}$$

(13) $$V_{2}=\frac{1}{2}e_{\omega }^{2}$$

According to Equations (6) and (7), the differential of $V_{1}$ and $V_{2}$ are

(14) $${\dot{V}}_{1}=e_{\theta }{\dot{e}}_{\theta }=e_{\theta }fosc\left(e_{\theta },\nu_{\theta 2},\sigma ,h\right)$$

(15) $${\dot{V}}_{2}=e_{\omega }{\dot{e}}_{\omega }=e_{\omega }fosc\left(e_{\omega },\nu_{\omega 2},\sigma ,h\right)$$

According to the characteristics of the fosc, *e* and $fosc\left(e,\nu_{2},\sigma ,h\right)$ symbols are opposite. It can be seen that $V_{1}>0$, $V_{2}>0$, ${\dot{V}}_{1}\leq 0$, ${\dot{V}}_{2}\leq 0$. Based on the Lyapunov stability theory, the improved TD is asymptotically stable.

#### 3.3.2. Stability Analysis of Improved ESO

According to the state Equation (5) and the ESO (9), the error state equation of the ESO can be obtain as follows.

(16) $$\begin{array}{c} \left\{\begin{array}{c} \varepsilon_{a1}=e_{z1}\\ {\dot{\varepsilon }}_{a1}=\varepsilon_{a2}={\dot{e}}_{z1}\\ {\dot{\varepsilon }}_{a2}=-\beta_{2}fal\left(\varepsilon_{a1},\alpha ,\delta \right)-\beta_{1}\varepsilon_{a2} \end{array}\right.\\ \left\{\begin{array}{c} \varepsilon_{b1}=e_{z2}\\ {\dot{\varepsilon }}_{b1}=\varepsilon_{b2}={\dot{e}}_{z2}\\ {\dot{\varepsilon }}_{b2}=-\beta_{4}fal\left(\varepsilon_{b1},\alpha ,\delta \right)-\beta_{3}\varepsilon_{b2} \end{array}\right.\\ \left\{\begin{array}{c} \varepsilon_{c1}=e_{z3}\\ {\dot{\varepsilon }}_{c1}=\varepsilon_{c2}={\dot{e}}_{z3}\\ {\dot{\varepsilon }}_{c2}=-\beta_{6}fal\left(\varepsilon_{c1},\alpha ,\delta \right)-\beta_{5}\varepsilon_{c2} \end{array}\right. \end{array}$$

Let the Lyapunov fucntion of the error state equation be:

(17) $$V_{3}=\int_{0}^{\varepsilon_{a1}}2\beta_{2}fal\left(\varepsilon ,\alpha ,\delta \right)d\varepsilon +\varepsilon_{a2}^{2}$$

(18) $$V_{4}=\int_{0}^{\varepsilon_{b1}}2\beta_{4}fal\left(\varepsilon ,\alpha ,\delta \right)d\varepsilon +\varepsilon_{b2}^{2}$$

(19) $$V_{5}=\int_{0}^{\varepsilon_{c1}}2\beta_{6}fal\left(\varepsilon ,\alpha ,\delta \right)d\varepsilon +\varepsilon_{c2}^{2}$$

For each ESO, there is at least one point $\chi_{a}\in \left[0,\varepsilon_{a1}\right]$, $\chi_{b}\in \left[0,\varepsilon_{b1}\right]$, $\chi_{c}\in \left[0,\varepsilon_{c1}\right]$, satisfying

(20) $$\begin{array}{cc} V_{3} & =\int_{0}^{\varepsilon_{a1}}2\beta_{2}fal\left(\varepsilon ,\alpha ,\delta \right)d\varepsilon +\varepsilon_{a2}^{2}\\ \; & =2\beta_{2}fal\left(\chi_{a},\alpha ,\delta \right)\varepsilon_{a1}+\varepsilon_{a2}^{2} \end{array}$$

(21) $$\begin{array}{cc} V_{4} & =\int_{0}^{\varepsilon_{b1}}2\beta_{4}fal\left(\varepsilon ,\alpha ,\delta \right)d\varepsilon +\varepsilon_{b2}^{2}\\ \; & =2\beta_{4}fal\left(\chi_{b},\alpha ,\delta \right)\varepsilon_{a1}+\varepsilon_{a2}^{2} \end{array}$$

(22) $$\begin{array}{cc} V_{5} & =\int_{0}^{\varepsilon_{c1}}2\beta_{6}fal\left(\varepsilon ,\alpha ,\delta \right)d\varepsilon +\varepsilon_{c2}^{2}\\ \; & =2\beta_{6}fal\left(\chi_{c},\alpha ,\delta \right)\varepsilon_{a1}+\varepsilon_{a2}^{2} \end{array}$$

As $fal\left(\varepsilon ,\alpha ,\delta \right)$ and ε are both positive and negative, for $\beta_{2}>0$, $\beta_{4}>0$, $\beta_{6}>0$, it can be derived $V_{3}>0$, $V_{4}>0$, $V_{5}>0$.

The derivatives of Equations (17)–(19) are

(23) $$\begin{array}{cc} {\dot{V}}_{3} & =2\varepsilon_{a2}\beta_{2}fal\left(\varepsilon_{a1},\alpha ,\delta \right)+2\varepsilon_{a2}{\dot{\varepsilon }}_{a2}\\ \; & =2\varepsilon_{a2}\beta_{2}fal\left(\varepsilon_{a1},\alpha ,\delta \right)+2\varepsilon_{a2}\left[-\beta_{2}fal\left(\varepsilon_{a1},\alpha ,\delta \right)-\beta_{1}\varepsilon_{a2}\right]\\ \; & =-2\beta_{1}\varepsilon_{a2}^{2} \end{array}$$

(24) $$\begin{array}{cc} {\dot{V}}_{4} & =2\varepsilon_{b2}\beta_{4}fal\left(\varepsilon_{b1},\alpha ,\delta \right)+2\varepsilon_{b2}{\dot{\varepsilon }}_{b2}\\ \; & =2\varepsilon_{b2}\beta_{4}fal\left(\varepsilon_{b1},\alpha ,\delta \right)+2\varepsilon_{b2}\left[-\beta_{4}fal\left(\varepsilon_{b1},\alpha ,\delta \right)-\beta_{3}\varepsilon_{b2}\right]\\ \; & =-2\beta_{3}\varepsilon_{b2}^{2} \end{array}$$

(25) $$\begin{array}{cc} {\dot{V}}_{5} & =2\varepsilon_{c2}\beta_{6}fal\left(\varepsilon_{c1},\alpha ,\delta \right)+2\varepsilon_{c2}{\dot{\varepsilon }}_{c2}\\ \; & =2\varepsilon_{c2}\beta_{6}fal\left(\varepsilon_{c1},\alpha ,\delta \right)+2\varepsilon_{c2}\left[-\beta_{6}fal\left(\varepsilon_{c1},\alpha ,\delta \right)-\beta_{5}\varepsilon_{c2}\right]\\ \; & =-2\beta_{5}\varepsilon_{c2}^{2} \end{array}$$

If the coeffcient satisfis that $\beta_{1}>0$, $\beta_{3}>0$, $\beta_{5}>0$, then it can be derived that ${\dot{V}}_{3}\leq 0$, ${\dot{V}}_{4}\leq 0$, ${\dot{V}}_{5}\leq 0$. According to the Lyapunov stability theory, the ESO is asymptotically stable.

#### 3.3.3. Stability Analysis of Improved NLSEF

Because the convergence of the improved ESO has been proved, it can be guaranteed that $z_{1}->x_{1}$, $z_{3}->x_{2}$, and $z_{5}->x_{3}$. The improved TD can guarantee that $v_{\theta 1}->\theta_{ref}$, $v_{\omega 2}->\omega_{ref}$, and $v_{\omega 2}->0$. According to Equations (5) and (11), the error state equation can be rewritten as follows:

(26) $$\begin{array}{c} \left\{\begin{array}{c} e_{a}=x_{1}-\theta_{ref}\approx -e_{c1}\\ e_{b}=x_{2}-\omega_{ref}\approx -e_{c2}\\ e_{c}=x_{3}\approx -e_{c3} \end{array}\right.\\ \left\{\begin{array}{c} {\dot{e}}_{a}={\dot{x}}_{1}-{\dot{\theta }}_{ref}=x_{2}\\ {\dot{e}}_{b}={\dot{x}}_{2}-{\dot{\omega }}_{ref}=x_{3}\\ {\dot{e}}_{c}={\dot{x}}_{3}=b_{0}u_{0}\\ \;\;\;=-b_{0}\zeta_{1}fal\left(e_{a},\alpha_{1},\delta \right)-b_{0}\zeta_{2}fal\left(e_{b},\alpha_{2},\delta \right)-b_{0}\zeta_{3}fal\left(e_{c},\alpha_{3},\delta \right) \end{array}\right. \end{array}$$

Let $f\left(e_{a}\right)=b_{0}\zeta_{1}fal\left(e_{a},\alpha_{1},\delta \right)$ and $g\left(e_{b}\right)=b_{0}\zeta_{2}fal\left(e_{b},\alpha_{2},\delta \right)$. There exists κ>0 satisfies that $0<a_{0}=b_{0}\zeta_{3}\frac{fal\left(e_{c},\alpha_{3},\delta \right)}{e_{c}}\leq a=b_{0}\zeta_{3}\kappa$. The Lyapunov function is selected as follows.

(27) $$\begin{array}{cc} V_{6} & =a_{0}\int_{0}^{e_{a}}f\left(x\right)dx+e_{b}f\left(e_{a}\right)+\int_{0}^{e_{b}}g\left(y\right)dy+\frac{1}{2}{\left(a_{0}e_{b}+e_{c}\right)}^{2}\\ \; & \leq a\int_{0}^{e_{a}}f\left(x\right)dx+e_{b}f\left(e_{a}\right)+\int_{0}^{e_{b}}g\left(y\right)dy+\frac{1}{2}{\left(ae_{b}+e_{c}\right)}^{2}\\ \; & =aF\left(e_{a}\right)+e_{b}f\left(e_{a}\right)+G\left(e_{b}\right)+\frac{1}{2}{\left(ae_{b}+e_{c}\right)}^{2}\\ \; & =\frac{{\left(2G\left(e_{b}\right)+e_{b}f\left(e_{a}\right)\right)}^{2}+4aF\left(e_{a}\right)G\left(e_{b}\right)-{e_{b}}^{2}f^{2}\left(e_{a}\right)}{4G\left(e_{b}\right)}+\frac{1}{2}{\left(ae_{b}+e_{c}\right)}^{2}\\ \; & =\frac{4a\int_{0}^{e_{a}}f\left(x\right)dx\int_{0}^{e_{b}}g\left(y\right)dy-4\int_{0}^{e_{a}}f\left(x\right)f^{\prime }\left(x\right)dx\int_{0}^{e_{b}}ydy}{4G\left(e_{b}\right)}+\frac{{\left(2G\left(e_{b}\right)+e_{b}f\left(e_{a}\right)\right)}^{2}}{4G\left(e_{b}\right)}+\frac{1}{2}{\left(ae_{b}+e_{c}\right)}^{2}\\ \; & =\frac{4\int_{0}^{e_{a}}f\left(x\right)\left[\int_{0}^{e_{b}}\left(ag\left(y\right)-f^{\prime }\left(y\right)e_{b}\right)dy\right]dx}{4G\left(e_{b}\right)}+\frac{{\left(2G\left(e_{b}\right)+e_{b}f\left(e_{a}\right)\right)}^{2}}{4G\left(e_{b}\right)}+\frac{1}{2}{\left(ae_{b}+e_{c}\right)}^{2} \end{array}$$

According to the characteristics of the fal, the equation satisfies $a\frac{g\left(e_{b}\right)}{e_{b}}-f^{\prime }\left(e_{a}\right)>0$ in case $e_{a}\neq 0$ and $e_{b}\neq 0$ , then $V_{6}>0$. The derivative of $V_{6}$ is

(28) $${\dot{V}}_{6}=af\left(e_{a}\right)e_{b}+f^{\prime }\left(e_{a}\right)e_{b}^{2}+f\left(e_{a}\right)e_{c}+g\left(e_{b}\right)e_{c}+\left(ae_{b}+e_{c}\right)\left(ae_{c}+{\dot{e}}_{c}\right)$$

Substituting ${\dot{e}}_{c}$ into Equation (28), it can be rewritten as follows.

(29) $${\dot{V}}_{6}=-\left(a\frac{g\left(e_{b}\right)}{e_{b}}-f^{\prime }\left(e_{a}\right)\right)e_{b}^{2}\leq 0$$

According to the Lyapunov stability theory, the improved NLSEF is asymptotically stable.

### 3.4. Parameters Tuning of Improved ADRC

Through the above content, the range of parameters could be obtained. To achieve a good control performance in the actual working process, the parameters of the controller need to be adjusted. There are 11 parameters that need to be adjusted (δ, $b_{0}$, $\beta_{1}$, $\beta_{2}$, $\beta_{3}$, $\beta_{4}$, $\beta_{5}$, $\beta_{6}$, $\zeta_{1}$, $\zeta_{2}$, $\zeta_{3}$). The tuning of controller parameters can be regarded as a multi-objective optimization issue, and the meta-heuristic algorithm performs well in this respect. The controller parameters are tuned by the slime mould algorithm based on a Levy flight operator (LF-SMA) in this paper.

#### 3.4.1. Slime Mould Algorithm based on a Levy Flight Operator (LF-SMA)

The LF-SMA is one of the high-performance meta-heuristic algorithms [37,38]. The algorithm consists of two parts.

Firstly, according to the fitness function and the position of slime mould *P*, the fitness function value and the corresponding weight *W* of each location can be calculated as follows:

(30) $$W\left(i\right)=\left\{\begin{array}{c} 1+r\cdot \log\left(\frac{bestFitness-Fitness\left(i\right)}{bestFitness-worstFitness}+1\right),Fitness\left(i\right)\geq middleFitness\\ 1-r\cdot \log\left(\frac{bestFitness-Fitness\left(i\right)}{bestFitness-worstFitness}+1\right),Fitness\left(i\right)<middleFitness \end{array}\right.$$

where *r* denotes the random value in the interval of [0,1].

Secondly, update the position based on the weight Equation (30). In this part, the Levy flight operator is added to enhance the global search performance of the algorithm. The formula for updating the position can be expressed as follows:

(31) $$P_{i}^{t+1}=\left\{\begin{array}{cc} r\cdot \left(UB-LB\right)+LB & ,0\leq r<z\\ P_{best}^{t}+vb\cdot \left(W\left(i\right)\cdot P_{A}^{t}-P_{B}^{t}\right)⊗Levy & ,z\leq r<p\\ vc\cdot P_{i}^{t} & ,p\leq r\leq 1 \end{array}\right.$$

where LB and UB are the lower and upper boundaries. $p=\tanh\left|Fitness\left(i\right)-DF\right|$. DF is the best fitness obtained in all iterations. vb is a parameter with a range of [−a,a], $a=arctanh\left(-\left(\frac{t}{\max\_t}\right)+1\right)$, vc decreases linearly from one to zero. $P_{best}^{t}$ represents the individual location with the best fitness value currently found, $P_{A}^{t}$ and $P_{B}^{t}$ represent two individuals randomly selected from slime mould. ⊗ is the Hadamard product. *z* is an adjustment parameter.

The Levy flight operator can be expressed as follows:

(32) $$\begin{array}{c} Levy\left(\alpha \right)∼\frac{\phi u}{{\left|u\right|}^{\frac{1}{\alpha }}}\\ \phi ={\left(\frac{\gamma \left(1+\alpha \right)\cdot \sin\left(\frac{\pi }{2}\cdot \alpha \right)}{\gamma \left(\frac{1+\alpha }{2}\right)\cdot \alpha \cdot 2^{\frac{\alpha -1}{2}}}\right)}^{\frac{1}{\alpha }} \end{array}$$

where γ is standard Gamma function, and α=1.5.

#### 3.4.2. Fitness Function

There are multiple ways to estimate the performance of the controller. The Integral of Time multiplied by Absolute Error (ITAE) is one of the most commonly used. In order to achieve precise tracking, the fitness function used in our scheme is as follows:

(33) $$ITAE=\int_{0}^{\infty }\left(w_{1}\left|e_{1}\right|+w_{2}\left|e_{2}\right|+w_{3}\left|e_{3}\right|\right)tdt+w_{4}\kappa$$

where $w_{i}$ is the weight factor, κ is the amount of overshoot.

**Figure 2:**
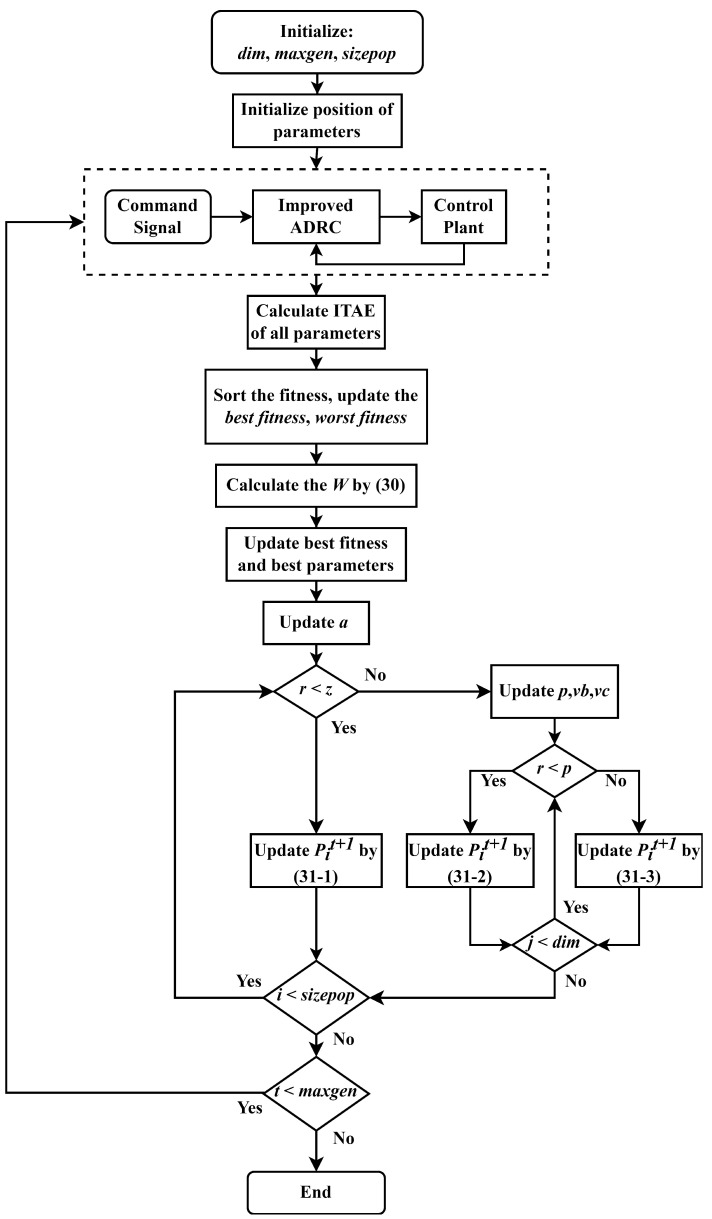
The flow chart of parameters tuning based on LF-SMA.

The parameters can be optimized after the fitness function is determined. When the fitness function reaches the minimum value, the corresponding parameters are the optimal parameters of the controller. The flow chart of the parameter tuning algorithm is shown in Figure 2.

## 4. Improved Harmonic Injection Method for Torque Ripple Suppression

ADRC can suppress the influence of the disturbances of the unknown parts inside and outside the system to a certain extent. In order to further improve the system accuracy, the harmonic injection module is added to the control scheme to suppress the torque ripple harmonics.

There is no phase difference between the harmonic component in the stator current and the back EMF of the BLDC motor, and the harmonic components are the same [39]. If the components of the back EMF harmonic are known, the torque ripple can be suppressed by injecting a specific harmonic current into the stator winding. The back EMF components can be expressed as follows.

(34) $$e_{abcs}=\omega_{r}\lambda_{0}\sum_{m=1}^{\infty }\left(2m-1\right)K_{2m-1}\left[\begin{array}{c} \cos\left(\left(2m-1\right)\theta_{r}\right)\\ \cos\left(\left(2m-1\right)\left(\theta_{r}-\frac{2\pi }{3}\right)\right)\\ \cos\left(\left(2m-1\right)\left(\theta_{r}+\frac{2\pi }{3}\right)\right) \end{array}\right]$$

The stator current can be written as follows.

(35) $$I_{abcs}=\sum_{n=0}^{\infty }\left(I_{6n+1}\left[\begin{array}{c} \cos\left(\left(6n+1\right)\theta_{r}\right)\\ \cos\left(\left(6n+1\right)\left(\theta_{r}-\frac{2}{3}\pi \right)\right)\\ \cos\left(\left(6n+1\right)\left(\theta_{r}+\frac{2}{3}\pi \right)\right) \end{array}\right]\right)+\sum_{n=1}^{\infty }\left(I_{6n-1}\left[\begin{array}{c} \cos\left(\left(6n-1\right)\theta_{r}\right)\\ \cos\left(\left(6n-1\right)\left(\theta_{r}-\frac{2}{3}\pi \right)\right)\\ \cos\left(\left(6n-1\right)\left(\theta_{r}+\frac{2}{3}\pi \right)\right) \end{array}\right]\right)$$

Because the motor winding is connected by wye type, the stator current harmonic components of the third harmonic and the third multiple harmonics are 0. Taking (35) into the torque expression, the further expression of the motor torque can be obtained.

(36) $$\begin{array}{cc} T_{e} & =\frac{P}{2}\lambda_{0}\sum_{m=1}^{\infty }\left(2m-1\right)K_{2m-1}{\left[\begin{array}{c} i_{as}\\ i_{bs}\\ i_{cs} \end{array}\right]}^{T}\left[\begin{array}{c} \cos\left(\left(2m-1\right)\theta_{r}\right)\\ \cos\left(\left(2m-1\right)\left(\theta_{r}-\frac{2\pi }{3}\right)\right)\\ \cos\left(\left(2m-1\right)\left(\theta_{r}+\frac{2\pi }{3}\right)\right) \end{array}\right]\\ \; & =\frac{P}{2}\lambda_{0}\left(\sum_{n=1}^{\infty }\sum_{m=1}^{\infty }\left(\left(2m-1\right)I_{6n-1}K_{2m-1}{\left[\begin{array}{c} \cos\left(\left(6n-1\right)\theta_{r}\right)\\ \cos\left(\left(6n-1\right)\left(\theta_{r}-\frac{2}{3}\pi \right)\right)\\ \cos\left(\left(6n-1\right)\left(\theta_{r}+\frac{2}{3}\pi \right)\right) \end{array}\right]}^{T}\left[\begin{array}{c} \cos\left(\left(2m-1\right)\theta_{r}\right)\\ \cos\left(\left(2m-1\right)\left(\theta_{r}-\frac{2}{3}\pi \right)\right)\\ \cos\left(\left(2m-1\right)\left(\theta_{r}+\frac{2}{3}\pi \right)\right) \end{array}\right]\right)\right.\\ \; & \left.+\sum_{n=0}^{\infty }\sum_{m=1}^{\infty }\left(\left(2m-1\right)I_{6n+1}K_{2m-1}{\left[\begin{array}{c} \cos\left(\left(6n+1\right)\theta_{r}\right)\\ \cos\left(\left(6n+1\right)\left(\theta_{r}-\frac{2}{3}\pi \right)\right)\\ \cos\left(\left(6n+1\right)\left(\theta_{r}+\frac{2}{3}\pi \right)\right) \end{array}\right]}^{T}\left[\begin{array}{c} \cos\left(\left(2m-1\right)\theta_{r}\right)\\ \cos\left(\left(2m-1\right)\left(\theta_{r}-\frac{2}{3}\pi \right)\right)\\ \cos\left(\left(2m-1\right)\left(\theta_{r}+\frac{2}{3}\pi \right)\right) \end{array}\right]\right)\right) \end{array}$$

After simplification, we can obtain the following equation.

(37) $$T_{e}=T_{0}+\sum_{n=1}^{\infty }T_{6n}\cos\left(6n\cdot \theta_{r}\right),n=1,2,3,\ldots$$

For the back EMF and stator current harmonic components, if only the 6th and the 12th harmonic components are retained, and the influence of high harmonic components is ignored, we can get (38).

(38) $$\left[\begin{array}{c} T_{0}\\ T_{6}\\ T_{12}\\ T_{18}\\ T_{24}\\ \ldots \end{array}\right]=\frac{3}{2}P\lambda_{0}K_{m}^{*}\left[\begin{array}{c} I_{1}\\ I_{5}\\ I_{7}\\ I_{11}\\ I_{13}\\ \ldots \end{array}\right]$$

$$K_{m}^{*}=\left[\begin{array}{ccccc} K_{1} & 5K_{5} & 7K_{7} & 11K_{11} & 13K_{13}\\ 5K_{5}+7K_{7} & K_{1}+11K_{11} & K_{1}+13K_{13} & 5K_{5} & 7K_{7}\\ 11K_{11}+13K_{13} & 7K_{7} & 5K_{5} & K_{1} & K_{1}\\ 0 & 13K_{13} & 11K_{11} & 7K_{7} & 5K_{5}\\ 0 & 0 & 0 & 13K_{13} & 11K_{11} \end{array}\right]$$

Because $I_{1}$ and $K_{1}$ are often 1–2 orders of magnitude higher than the high-order harmonic components, which will lead to the results that the torque harmonic ripples higher than 18 times will be far less than the 6th and the 12th. So, in most cases, we only need to consider the 6th and the 12th harmonic components. At the same time, the increase of high-order harmonic currents often enhances the equivalent resistance of the motor winding, thus increasing copper consumption. Therefore, in the control strategy, the increase of high-order harmonic currents should be avoided.

When the harmonic of high order torque ripple is zero, there is no torque ripple in electromagnetic torque. Then (38) can be rewritten as (39). The corresponding reference currents are given in Appendix A (Scheme A).

(39) $$\left[\begin{array}{c} T_{0}\\ 0\\ 0\\ 0\\ 0 \end{array}\right]=\frac{3}{2}P\lambda_{0}K_{m}^{*}\left[\begin{array}{c} I_{1}\\ I_{5A}^{*}\\ I_{7A}^{*}\\ I_{11A}^{*}\\ I_{13A}^{*} \end{array}\right]$$

Furthermore, in order to study the effect of changing injection harmonic current components on torque ripple, two additional harmonic injection schemes (Scheme B, scheme C) are proposed. Scheme B defaults that $I_{11}=0$ and $I_{13}=0$. Scheme C defaults that $K_{11}=0$, $K_{13}=0$, $I_{11}=0$ and $I_{13}=0$.

The corresponding reference currents are given in Appendix B (Scheme B), Appendix C (Scheme C).

## 5. Experiment Results

**Figure 3:**
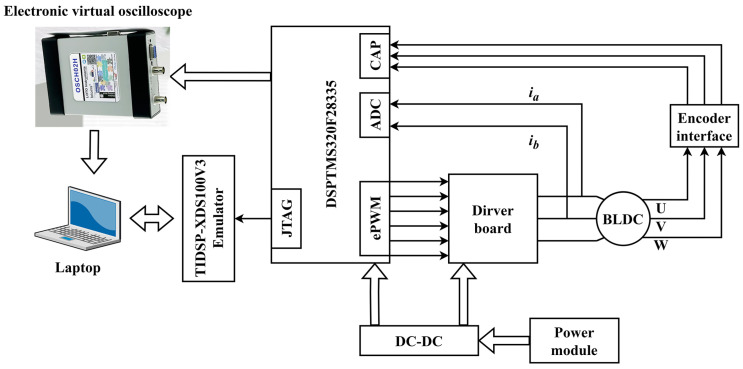
The principle block diagram of experimental setup for the BLDC motor drive system.

**Figure 4:**
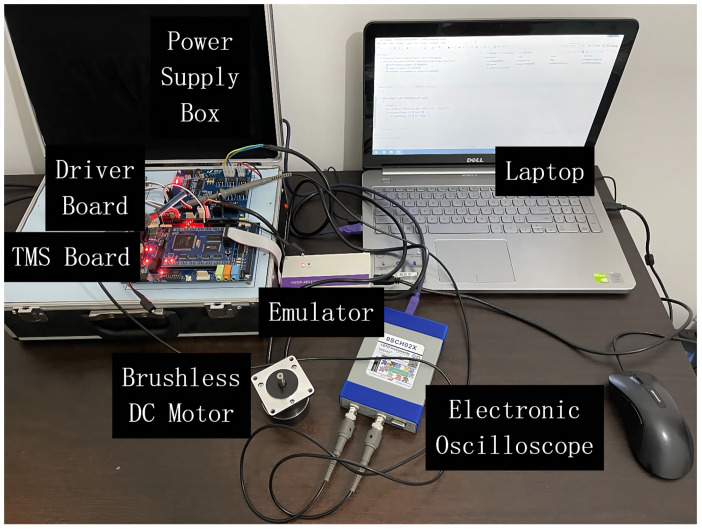
Experiment platform of the BLDC motor drive system.

In order to test the performance of the control method, the experiment platform is established. The BLDC motor servo system mainly contains a BLDC motor, a power module, a TMS320F28335 control board, a driver board including a voltage source inverter, a position detection module, etc. The parameters of the BLDC motor are listed in Table 1. The principle block diagram of experimental setup and the experimental platform are shown in Figure 3 and Figure 4.

**Figure 5:**
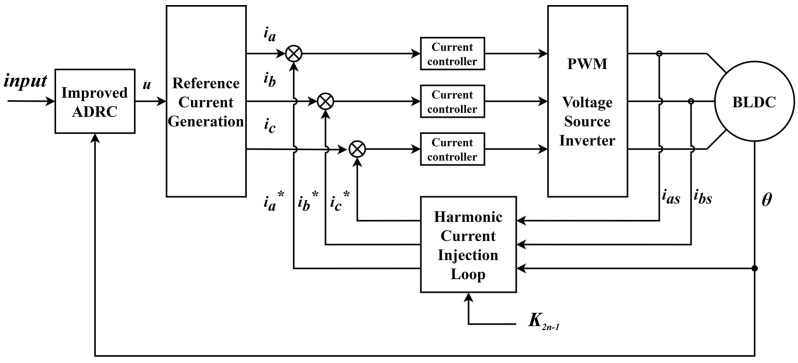
The principle block diagram of the harmonic injection ADRC method.

**Figure 6:**
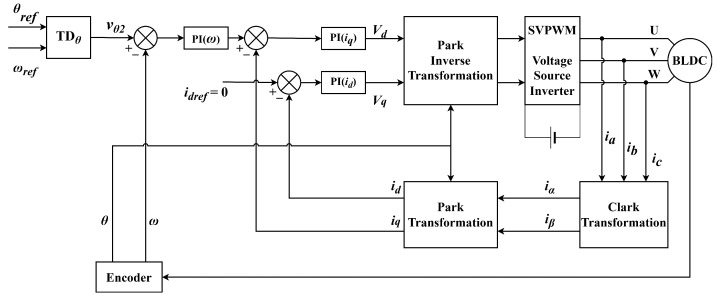
The principle block diagram of the PI + FOC method.

The comparison control method is based on the PI + FOC scheme mentioned in Reference [29]. The principle block diagram of the harmonic injection ADRC method and PI + FOC method are shown in Figure 5 and Figure 6. The control parameters of the controllers are listed in Table 2.

### 5.1. Test of Working Mode Switch and Positioning Performance

**Figure 7:**
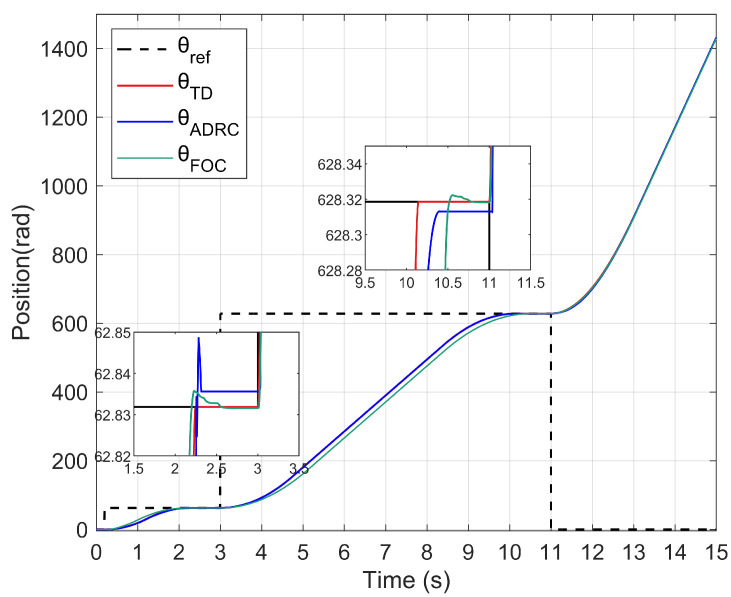
The actual position of the improved ADRC system and the PI + FOC system.

In order to validate the performance of working mode switch, the following input signals are used as the given input to the system. As shown in Figure 7, the given input signals change twice during the operation of the system. Start with $\theta_{ref}$ = 20 π, $\omega_{ref}=0$ as the input, change the input signals to $\theta_{ref}$ = 200 π, $\omega_{ref}$ = 1000 rpm in the third second and change the input signals to $\theta_{ref}$ = 0, $\omega_{ref}$ = 2500 rpm in the 11th second. According to the experimental outcomes, the positioning performance indexes of the control scheme can be acquired, as shown in Table 3.

The positioning errors in Table 3 indicate cumulative errors over the rise periods. The experimental results confirm that the proposed ADRC control scheme capable of the transformation of working mode and tracking the given signals effectively. Compared with PI + FOC method, the improved ADRC scheme is slightly deficient in positioning performance.

### 5.2. Test of Speed Stability and Torque Ripple Suppression Performance

**Figure 8:**
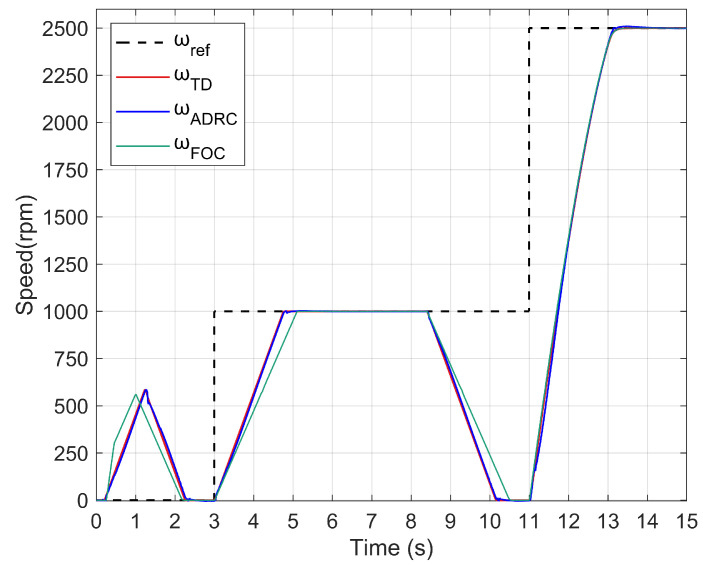
The actual speed of the improved ADRC system and the PI + FOC system.

In order to test the speed performance of the control scheme, the improved ADRC with harmonic injection schemes are tested. The actual speed of the systems based on the given input in Section 5.1 are shown in Figure 8.

**Figure 9:**
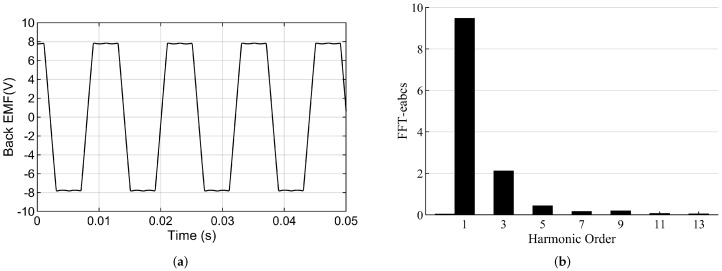
(**a**) The back EMF of the motor in 2500 rpm (**b**) FFT of the back EMF.

Figure 9a,b show the back EMF waveform and the FFT waveform of the BLDC motor at 2500 rpm, respectively. The harmonic content of the back EMF is listed in Table 4.

**Figure 10:**
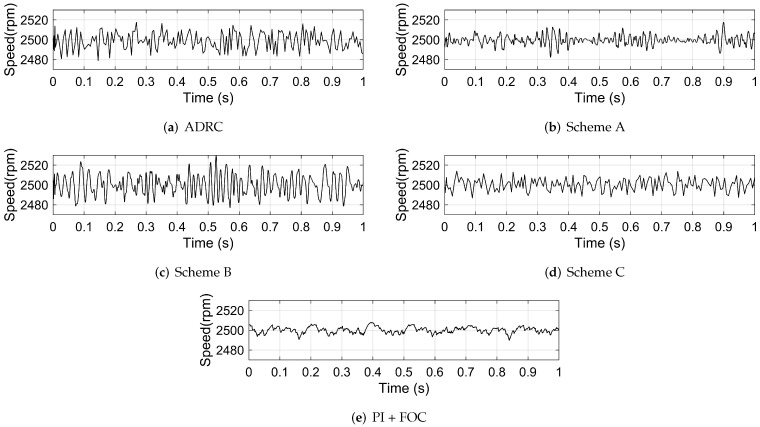
The actual speed of the system based on: (**a**) ADRC; (**b**) scheme A; (**c**) scheme B; (**d**) scheme C; (**e**) PI + FOC.

**Figure 11:**
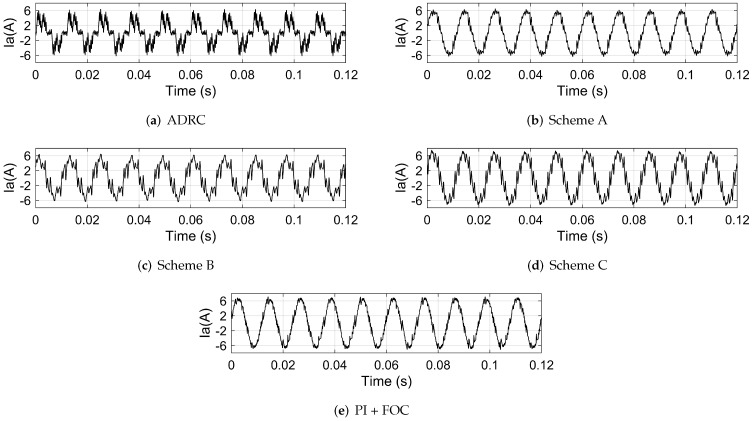
The motor phase current of the system based on: (**a**) ADRC; (**b**) scheme A; (**c**) scheme B; (**d**) scheme C; (**e**) PI + FOC.

**Figure 12:**
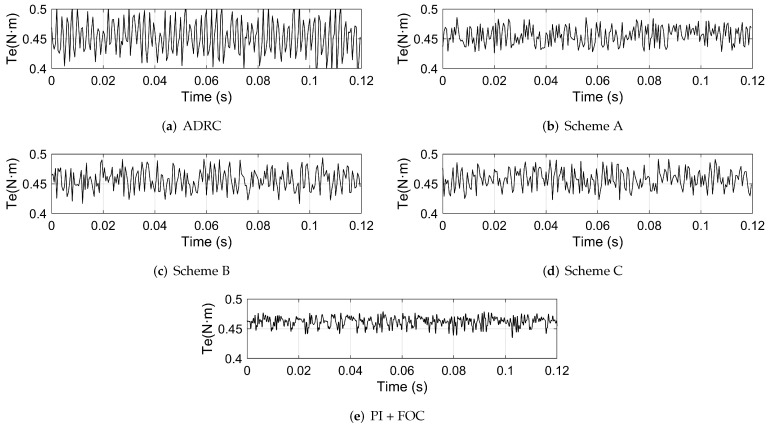
The motor torque of the system based on: (**a**) ADRC; (**b**) scheme A; (**c**) scheme B; (**d**) scheme C; (**e**) PI + FOC.

**Figure 13:**
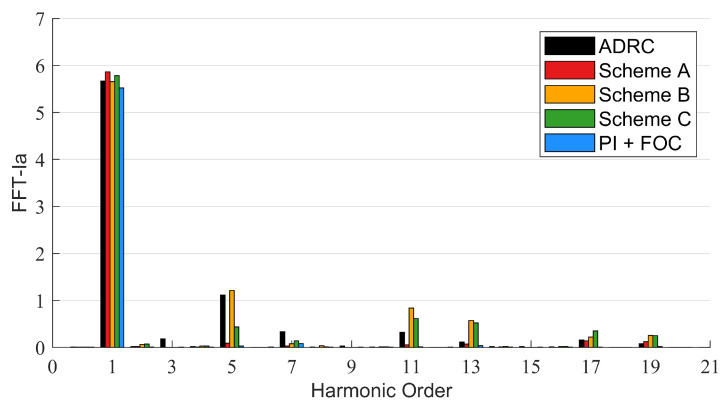
FFT of motor phase current.

**Figure 14:**
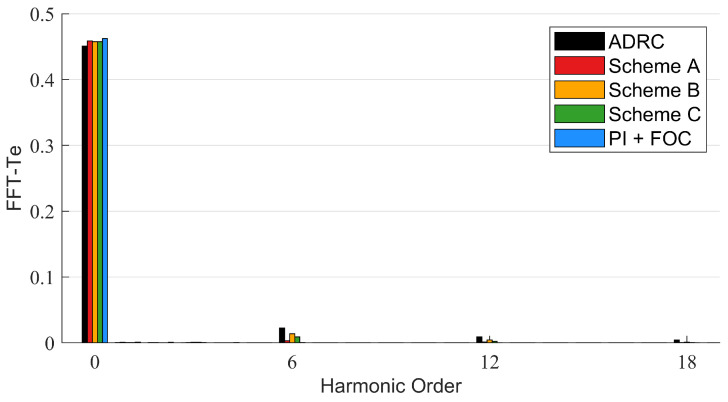
FFT of motor torque.

The disturbance torque is added by setting a 0.1 N · m load. The actual speed, the motor phase current and the motor torque of the system based on different control schemes are shown in Figure 10, Figure 11 and Figure 12, respectively. The FFT of the motor phase current and torque is shown in Figure 13 and Figure 14, and the harmonic components are listed in Table 5 and Table 6.

In order to comprehensively assess the performance of the control schemes, the Total Harmonic Distortion $\left(THD_{i}\right)$ of the motor phase current and Ripple Factor $\left(RF_{T}\right)$ of motor torque are listed in Table 7. $THD_{i}$ and $RF_{T}$ are calculated as follows.

(40) $$THD_{i}=\frac{\sqrt{{\left|I_{5}\right|}^{2}+{\left|I_{7}\right|}^{2}+{\left|I_{11}\right|}^{2}+{\left|I_{13}\right|}^{2}+{\left|I_{17}\right|}^{2}+{\left|I_{19}\right|}^{2}}}{I_{1}}$$

(41) $$RF_{T}=\frac{\sqrt{{T_{6}}^{2}+{T_{12}}^{2}+{T_{18}}^{2}}}{T_{0}}$$

The above experimental results reveal that the improved harmonic injection ADRC method can successfully suppress torque harmonic ripple and improve speed stability of the system. The high harmonic orders of torque ripple lead to a little influence on the speed precision of BLDCM, so they could not be considered in the practical control.

## 6. Conclusions

Based on the working requirements of OMSS, an improved harmonic injection ADRC scheme is proposed in this paper, which can realize the conversion between ‘point-to-point control’ and ‘speed stability control’ without changing the parameters of ADRC. In order to validate the efficiency of the control scheme, an experimental platform is developed to examine the positioning performance, speed stability and torque ripple suppression. The experimental outcomes reveal that the high harmonic orders of torque ripple lead to a little influence on the speed precision of BLDCM, so they could not be considered in the practical control. The addition of the harmonic injection scheme effectively reduces the torque ripple and improves the speed stability. It is worth noting that the stator current acquisition in the harmonic injection scheme is completed in the stationary reference frame. Compared with PI + FOC control, the control scheme proposed realizes considerable performance with a low precision encoder in the harmonic injection scheme, and it only needs one current sensor to realize the system control. It also improves the computational efficiency and saves the cost of the system. However, the control scheme has limited control performance for high-power systems with high rotational speed.

In future work, we will consider adopting appropriate algorithms to realize the adaptive tuning of the controller parameters to ensure the stability of the system under complex working conditions. Furthermore, the stability of the proposed ADRC scheme will be further discussed in the frequency domain. In addition, the flight test of the control scheme will be carried out based on the OMSS platform of the ASHIS, and the effectiveness of the control scheme will be verified in a more complex working environment. Further, we also consider combining the proposed control scheme with the Kalman filter to realize the matching of images and image points in ASHIS.

## Figures and Tables

**Table 1:** BLDC motor parameters.

Parameters	Value
Pole	4
Rated voltage	24 V
Rated speed	3000 rpm
Moment of inertia	120 g/cm^2^
Resistance	0.6 Ω
Inductors	0.75 mH
Back EMF constant	6.23 V/krpm
Torque constant	0.065 N · m/A
Encoder	2000

**Table 2:** The control parameters of the controllers.

Controller	Parameters											
Improved ADRC	$\sigma_{1}$			$\sigma_{2}$			δ			$b_{0}$		
	80			3000			6.11			6.23		
	α			$\alpha_{1}$			$\alpha_{2}$			$\alpha_{3}$		
	0.5			0.5			0.25			0.25		
	$\beta_{1}$		$\beta_{2}$		$\beta_{3}$		$\beta_{4}$		$\beta_{5}$		$\beta_{6}$	
	6122.11		105,717.12		4672.55		156,806.31		9808.65		18,611.81	
	$\zeta_{1}$				$\zeta_{2}$				$\zeta_{3}$			
	8.62				4.68				0.21			
PI + FOC	$K_{p}$		$K_{i}$		$K_{pi_{d}}$		$K_{ii_{d}}$		$K_{pi_{q}}$		$K_{ii_{q}}$	
	0.12		1.11		10		600		20		600	

**Table 3:** Performance indexes of improved ADRC and PI + FOC.

	Working Mode	Rise Time (s)	Rise Positioning Error (rad)	Overshoot (rad)
Improved ADRC	0–20 π	2.26	0.0037	0.0178
	20–200 π	7.39	−0.0060	−0.0060
PI + FOC	0–20 π	2.20	0.0004	0.0038
	20–200 π	7.52	−0.0010	0.0030

**Table 4:** The harmonic contents of motor back EMF.

Harmonic Order	Harmonic Coefficient
1	1
3	−0.2216
5	0.0456
7	−0.0195
9	0.0216
11	−0.0089
13	0.0047

**Table 5:** Harmonic components of the motor phase current under different control schemes.

Control Scheme	$\left|I_{1}\right|$(A)	$\left|I_{5}\right|$(A)	$\left|I_{7}\right|$(A)	$\left|I_{11}\right|$(A)	$\left|I_{13}\right|$(A)	$\left|I_{17}\right|$(A)	$\left|I_{19}\right|$(A)
ADRC	5.6674	1.1139	0.3357	0.3238	0.1173	0.0158	0.0776
Scheme A	5.8642	0.0899	0.0297	0.0562	0.0751	0.1435	0.1232
Scheme B	5.6580	1.2142	0.0788	0.8393	0.5722	0.2229	0.2552
Scheme C	5.7828	0.4378	0.1438	0.6144	0.5226	0.3554	0.2500
PI + FOC	5.5224	0.0331	0.0842	0.0162	0.0401	0.0071	0.0181

**Table 6:** Harmonic components of the motor torque under different control schemes.

Control Scheme	$T_{0}$	$T_{6}$		$T_{12}$		$T_{18}$	
	(N · m)	(N · m)	(%)	(N · m)	(%)	(N · m)	(%)
ADRC	0.4511	0.0226	5.01	0.0091	2.02	0.0044	0.98
Scheme A	0.4588	0.0034	0.74	0.0013	0.28	0.0004	0.08
Scheme B	0.4574	0.0140	3.06	0.0045	0.98	0.0009	0.20
Scheme C	0.4576	0.0089	1.94	0.0021	0.46	0.0005	0.11
PI + FOC	0.4623	0.0004	0.09	0.00008	0.02	0.00005	0.01

**Table 7:** *THD*_*i*_ of the stator current and *RF*_*T*_ of motor torque.

	*THD*_*i*_	*RF*_*T*_
ADRC	0.2145	0.0549
Scheme A	0.0394	0.0079
Scheme B	0.2865	0.0322
Scheme C	0.1773	0.0201
PI + FOC	0.0185	0.0009

## Abbreviations

The following abbreviations are used in this manuscript:

ADRC	Active Disturbance Rejection Control
ASHIS	Airborne Scanning Hyperspectral Imaging Spectrometer
BLDC	Brushless DC
EMF	Electromotive Force
ESO	Extended State Observer
FOC	Field Oriented Control
FFT	Fast Fourier Transform
ILC	Iterative Learning Control
ITAE	Integral of Time multiplied by Absolute Error
LB	Lower Boundaries
LF-SMA	Slime Mould Algorithm based on a Levy Flight Operator
NLSEF	Nonlinear State Error Feedback
OMSS	Optomechanically Scanned System
PI	Proportional Integral
PID	Proportional Integral Derivative
RC	Repetitive ControL
RESO	Resonant Extended State Observer
RF	Ripple Factor
RPM	Revolutions Per Minute
TD	Tracking Differentiator
THD	Total Harmonic Distortion
UB	Upper Boundaries

## Appendix A. Harmonic Injection Scheme A

$$\left[\begin{array}{c} T_{0}\\ 0\\ 0\\ 0\\ 0 \end{array}\right]=\frac{3}{2}P\lambda_{0}\left[\begin{array}{ccccc} K_{1} & 5K_{5} & 7K_{7} & 11K_{11} & 13K_{13}\\ 5K_{5}+7K_{7} & K_{1}+11K_{11} & K_{1}+13K_{13} & 5K_{5} & 7K_{7}\\ 11K_{11}+13K_{13} & 7K_{7} & 5K_{5} & K_{1} & K_{1}\\ 0 & 13K_{13} & 11K_{11} & 7K_{7} & 5K_{5}\\ 0 & 0 & 0 & 13K_{13} & 11K_{11} \end{array}\right]\left[\begin{array}{c} I_{1}\\ I_{5A}^{*}\\ I_{7A}^{*}\\ I_{11A}^{*}\\ I_{13A}^{*} \end{array}\right]$$

$$\begin{array}{c} K_{A}^{*}=K_{1}^{2}{\left(11K_{11}-13K_{13}\right)}^{2}+2\times \left(7K_{7}\cdot 11K_{11}-5K_{5}\cdot 13K_{13}\right)\left(-5K_{5}\cdot 11K_{11}+7K_{7}\cdot 13K_{13}\right)\\ +K_{1}\left({\left(7K_{7}\right)}^{2}\cdot 11K_{11}+{\left(5K_{5}\right)}^{2}\cdot 13K_{13}+\left(-5K_{5}\cdot 7K_{7}+{\left(11K_{11}-13K_{13}\right)}^{2}\right)\left(11K_{11}+13K_{13}\right)\right) \end{array}$$

$$\begin{array}{c} {I_{5A}}^{*}=\frac{I_{1}}{{K_{A}}^{*}}\left(11K_{11}\left(5K_{5}\cdot 7K_{7}\left(5K_{5}+7K_{7}\right)-11K_{11}\cdot K_{1}\left(5K_{5}+2\times 7K_{7}\right)+5K_{5}{\left(11K_{11}\right)}^{2}\right)\right.\\ \left.-13K_{13}\left({\left(5K_{5}\right)}^{2}\left(5K_{5}+7K_{7}\right)-2\times K_{1}\cdot 5K_{5}\cdot 11K_{11}+{\left(11K_{11}\right)}^{2}\left(-5K_{5}+2\times 7K_{7}\right)\right)\right.\\ \left.+{\left(13K_{13}\right)}^{2}\left(K_{1}\cdot 5K_{5}+11K_{11}\left(5K_{5}-2\cdot 7K_{7}\right)\right)+5K_{5}\cdot {\left(13K_{13}\right)}^{3}\right) \end{array}$$

$$\begin{array}{c} {I_{7A}}^{*}=\frac{I_{1}}{{K_{A}}^{*}}\left(7K_{7}\cdot 11K_{11}\left(-7K_{7}\left(5K_{5}+7K_{7}\right)+11K_{11}\left(K_{1}+11K_{11}\right)\right)\right.\\ \left.+13K_{13}\left(5K_{5}\cdot 7K_{7}\left(5K_{5}+7K_{7}\right)+2\times K_{1}\cdot 7K_{7}\cdot 11K_{11}+{\left(11K_{11}\right)}^{2}\left(-2\times 5K_{5}+7K_{7}\right)\right)\right.\\ \left.-{\left(13K_{13}\right)}^{2}\left(K_{1}\left(2\times 5K_{5}+7K_{7}\right)+11K_{11}\left(2\times 5K_{5}-7K_{7}\right)\right)+7K_{7}\cdot {\left(13K_{13}\right)}^{3}\right) \end{array}$$

$$\begin{array}{c} {I_{11A}}^{*}=\frac{I_{1}}{{K_{A}}^{*}}\left(11K_{11}\left({\left(7K_{7}\right)}^{2}\cdot 11K_{11}+5K_{5}\cdot 7K_{7}\left(11K_{11}-13K_{13}\right)-{\left(5K_{5}\right)}^{2}\cdot 13K_{13}\right.\right.\\ \left.\left.-\left(K_{1}+11K_{11}+13K_{13}\right)\left({\left(11K_{11}\right)}^{2}-{\left(13K_{13}\right)}^{2}\right)\right)\right) \end{array}$$

$$\begin{array}{c} {I_{13A}}^{*}=\frac{I_{1}}{{K_{A}}^{*}}\left(-13K_{13}\left({\left(7K_{7}\right)}^{2}\cdot 11K_{11}+5K_{5}\cdot 7K_{7}\left(11K_{11}-13K_{13}\right)-{\left(5K_{5}\right)}^{2}\cdot 13K_{13}\right.\right.\\ \left.\left.-\left(K_{1}+11K_{11}+13K_{13}\right)\left({\left(11K_{11}\right)}^{2}-{\left(13K_{13}\right)}^{2}\right)\right)\right) \end{array}$$

## Appendix B. Harmonic Injection Scheme B

$$\left[\begin{array}{c} T_{0}\\ 0\\ 0 \end{array}\right]=\frac{3}{2}P\lambda_{0}\left[\begin{array}{ccc} K_{1} & 5K_{5} & 7K_{7}\\ 5K_{5}+7K_{7} & K_{1}+11K_{11} & K_{1}+13K_{13}\\ 11K_{11}+13K_{13} & 7K_{7} & 5K_{5} \end{array}\right]\left[\begin{array}{c} I_{1}\\ I_{5B}^{*}\\ I_{7B}^{*} \end{array}\right]$$

$$I_{5B}^{*}=\frac{I_{1}\left(-5K_{5}\left(5K_{5}+7K_{7}\right)+\left(K_{1}+13K_{13}\right)\left(11K_{11}+13K_{13}\right)\right)}{5K_{5}\left(K_{1}+11K_{11}\right)-7K_{7}\left(K_{1}+13K_{13}\right)}$$

$$I_{7B}^{*}=\frac{I_{1}\left(7K_{7}\left(5K_{5}+7K_{7}\right)-\left(K_{1}+11K_{11}\right)\left(11K_{11}+13K_{13}\right)\right)}{5K_{5}\left(K_{1}+11K_{11}\right)-7K_{7}\left(K_{1}+13K_{13}\right)}$$

## Appendix C. Harmonic Injection Scheme C

$$\left[\begin{array}{c} T_{0}\\ 0\\ 0 \end{array}\right]=\frac{3}{2}P\lambda_{0}\left[\begin{array}{ccc} K_{1} & 5K_{5} & 7K_{7}\\ 5K_{5}+7K_{7} & K_{1} & K_{1}\\ 0 & 7K_{7} & 5K_{5} \end{array}\right]\left[\begin{array}{c} I_{1}\\ I_{5C}^{*}\\ I_{7C}^{*} \end{array}\right]$$

$$I_{5C}^{*}=-\frac{5K_{5}\left(5K_{5}+7K_{7}\right)}{K_{1}\left(5K_{5}-7K_{7}\right)}I_{1}$$

$$I_{7C}^{*}=\frac{7K_{7}\left(5K_{5}+7K_{7}\right)}{K_{1}\left(5K_{5}-7K_{7}\right)}I_{1}$$
